# The Relationship between Processing Speed and Regional White Matter Volume in Healthy Young People

**DOI:** 10.1371/journal.pone.0136386

**Published:** 2015-09-23

**Authors:** Daniele Magistro, Hikaru Takeuchi, Keyvan Kashkouli Nejad, Yasuyuki Taki, Atsushi Sekiguchi, Rui Nouchi, Yuka Kotozaki, Seishu Nakagawa, Carlos Makoto Miyauchi, Kunio Iizuka, Ryoichi Yokoyama, Takamitsu Shinada, Yuki Yamamoto, Sugiko Hanawa, Tsuyoshi Araki, Hiroshi Hashizume, Yuko Sassa, Ryuta Kawashima

**Affiliations:** 1 Department of Functional Brain Imaging, Institute of Development, Aging and Cancer, Tohoku University, Sendai, Japan; 2 Division of Developmental Cognitive Neuroscience, Institute of Development, Aging and Cancer, Tohoku University, Sendai, Japan; 3 Devision of Meidcal Neuroimaging Analysis, Department of Community Medical Supports, Tohoku Medical Megabank Organization, Tohoku University, Sendai, Japan; 4 Department of Radiology and Nuclear Medicine, Institute of Development, Aging and Cancer, Tohoku University, Sendai, Japan; 5 Human and Social Response Research Division, International Research Institute of Disaster Science, Tohoku University, Sendai, Japan; 6 Smart Ageing International Research Center, Institute of Development, Aging and Cancer, Tohoku University, Sendai, Japan^7^Japan Society for the Promotion of Science, Tokyo, Japan; Banner Alzheimer's Institute, UNITED STATES

## Abstract

Processing speed is considered a key cognitive resource and it has a crucial role in all types of cognitive performance. Some researchers have hypothesised the importance of white matter integrity in the brain for processing speed; however, the relationship at the whole-brain level between white matter volume (WMV) and processing speed relevant to the modality or problem used in the task has never been clearly evaluated in healthy people. In this study, we used various tests of processing speed and Voxel-Based Morphometry (VBM) analyses, it is involves a voxel-wise comparison of the local volume of gray and white, to assess the relationship between processing speed and regional WMV (rWMV). We examined the association between processing speed and WMV in 887 healthy young adults (504 men and 383 women; mean age, 20.7 years, SD, 1.85). We performed three different multiple regression analyses: we evaluated rWMV associated with individual differences in the simple processing speed task, word–colour and colour–word tasks (processing speed tasks with words) and the simple arithmetic task, after adjusting for age and sex. The results showed a positive relationship at the whole-brain level between rWMV and processing speed performance. In contrast, the processing speed performance did not correlate with rWMV in any of the regions examined. Our results support the idea that WMV is associated globally with processing speed performance regardless of the type of processing speed task.

## Introduction

Processing speed is an individual cognitive ability measured by how fast an individual executes cognitive tasks, particularly elementary cognitive tasks [1], [2]. Moreover, processing speed is viewed as an overall measure of cognitive mechanisms that are widely used to support fluent execution of perceptual, cognitive and motor processes [3]. Indeed, processing speed is considered a key cognitive resource [4], [5], similar to attention, working memory and inhibition, underlying performance in various cognitive domains [2], [6], [7]. Accordingly, previous research showed that processing speed correlates with performance in various cognitive domains [4], [5], [8], [9], [10].

From the perspective of neuroscience, processing speed performance has traditionally been assumed to depend to a large extent on the properties of white matter [11], [12], [13]; the latter affects the speed of neural transmission. In fact, white matter includes all myelinated axons in the cerebrum, and the thickness of the myelin sheath is related to nerve conduction velocity; therefore, its relation to processing speed seems logical [11][14].

Previous neuroimaging studies have been focused on the two major structural properties of white matter: fractional anisotropy (FA), which reflects structural integrity of white matter[15], [16], in diffusion tensor imaging [5], [17], [18] and white matter volume (WMV) [11], [19], [20].

Previous studies of fractional anisotropy indicate that processing speed performance correlates with properties of regional white matter in brain areas relevant to the task. Processing speed is associated with visual choice reaction time in visual-related areas (in the number of posterior regions of the right hemisphere), the left middle frontal gyrus and the occipital and parietal areas during the digit–symbol task (fronto–parieto–occipital areas perform important functions in the execution of this task) [18], [21]. Moreover, the temporal lobe is associated with processing speed in the auditory reaction time task [22]. Conversely, there are studies that show a global association between processing speed tasks and white matter structure [17]; in fact, processing speed is related to white matter average FA of the whole brain [23].

Other studies indicate that processing speed performance correlates with total WMV (tWMV); in fact, processing speed is genetically related to tWMV[11]. There is also a relationship between reduced tWMV and impaired processing speed performance in patients with temporal lobe epilepsy [24].

Many studies have been focused on correlations between white matter integrity and processing speed in various tasks using diffusion tensor imaging [17], [18], [25], [26], [27]. Usually, studies on WMV involve specific populations, such as old adults [17–27], or specific diseases, such as temporal lobe epilepsy [24] or left-hemisphere stroke [5]. Additionally, previous anatomical studies did not verify the significance of regional WMV (rWMV), and this is one of the purposes of this study. To the best of our knowledge, no previous studies have observed a relationship between WMV and processing speed in a large sample of healthy young people. This study aimed to identify the rWMV correlates of processing speed in healthy young people. As we previously discussed [28], we consider that 1) FA and rWMV are moderately to weakly related, 2) the associations between them seem particularly weak in deep white matter areas [29] and 3) rWMV is known to significantly correlate with cognitive functions. Particularly, our previous studies that concurrently explored rWMV and FA correlates of cognitive functions showed more significant results of rWMV analyses in regions congruent with our hypothesis [30], [31]. In this study, we focus on rWMV correlates of processing speed using various tests of processing speed and Voxel-Based Morphometry (VBM) analyses that involves a voxel-wise comparison of the local volume of gray and white matter.

Based on previous studies, we are evaluating two sets of relatively opposite assumptions: 1) processing speed correlates with white matter structure globally; therefore it is rather problem independent and 2) processing speed correlates with white matter structure relevant to the modality or problem used in the task. Considering the contribution of processing speed to human cognitive activity, it is important to investigate the rWMV correlates with processing speed tasks in healthy adults compared with other neuroimaging methods. In fact, rWMV is widely accepted as the basis of individual intellectual abilities; the networks that underlie intellectual abilities can be identified by measuring WMV [32], [33], [34].

## Materials and Methods

### Subjects

Eight hundred eighty-seven healthy right-handed individuals (504 men and 383 women; mean age, 20.7 years, SD, 1.85 years) participated in this study as part of our ongoing project to explore the associations among brain imaging, cognitive functions, ageing, genetics and daily habits. All subjects were college students from Tohoku University in Japan. All subjects were undergraduate or postgraduate university students. All had normal vision and none had a history of neurological or psychiatric illness. None reported recent use of any psychoactive drugs or drugs that would be likely to negatively impact their cognitive abilities. The history of psychiatric illnesses and recent drug use were assessed using our laboratory’s routine questionnaire, in which each subject answered questions related to their current or previous experience with any of a list of illnesses and listed drugs they had taken recently. Handedness was evaluated using the Edinburgh Handedness Inventory [35]. The Ethics Committee of Tohoku University approved all procedures. Written informed consent was obtained from each subject for the projects in which they participated.

### Psychological tests

For the measurement of processing speed we used three tasks. Moreover, we measured general intelligence and verbal and spatial working memory of the participant, to verified the relationship between processing speed and more complicate cognitive tasks. All psychological tests were performed in the same day, with 15 minutes break between each tests; after another hour of rest, we performed the MRI scan.

#### Simple processing speed tasks

The Tanaka B-type intelligence test (TBIT) [36] type 3B was used for the measurement of processing speed. TBIT is a simple test for measuring simple processing speed. In all subtests, the subjects had to solve as many problems as possible within a certain period (a few minutes). This factor involved three subtests: a displacement task [substitute a figure (nine figures) with a number (1–9) according to a model chart], identification versus same–different judgments (Japanese kana characters; decide whether a pair of meaningless Japanese strings are the same) and marking of figures [select shapes identical to three samples from a series (sequence) of eight different shapes]. These tasks do not require recognition of words, and instead require recognition of symbols, letters, numbers and the like. These tasks do not involve complex cognitive processes but constitute simple processing speed tasks.

#### Word–colour and colour–word tasks (processing speed tasks with words)

The Stroop [37] task is a widely used paradigm in psychology and clinical practice [38]. During Stroop paradigms, subjects experience cognitive interference when they resolve interferences, for example, identifying the ink colour of a printed word while ignoring the word’s identity [37]. As in a previous study [39], [40], we used Hakoda’s version of the Stroop task [41]. This version of the Stroop task is of the matching type, requiring subjects to choose and check correct answers as possible from five options in 1 min, unlike the traditional oral naming task. This type of task is rather similar to the button-pressing matching-type Stroop tasks used in neuroimaging studies [42]. The task consists of two control tasks, a word–colour task and a colour–word task and a reverse Stroop task and a Stroop task. In this study, we focused on processing speed and used the normalised sum of the word–colour task and the colour–word task. These tasks require recognition of words.

#### Simple speed tasks

Our simple arithmetic task is similar to that constructed by Grabner et al. [43]. This task measures multiplication performance consisting of two forms of one-digit times one-digit multiplication problems (a simple arithmetic task with numbers between 2 and 9). The two forms of each task are the same, but the numbers used in the problems are different. Each form of the simple arithmetic task is presented with a 30-s time limit. The average of the performance on two forms was used. This task requires simple arithmetic calculations.

#### Raven's Advanced Progressive Matrix

Raven's Advanced Progressive Matrix (RAPM) [44], which is a psychometric measure of general intelligence [44], was used to assess general intelligence. The test contains 36 nonverbal items requiring fluid reasoning ability. Each item consists of a 3×3 matrix with a missing piece to be completed by selecting the best of 8 alternatives. The score of this test (number of correct answers in 30 min) was used as a psychometric index of individual intelligence. The RAPM was administered in a group setting in this study. The RAPM tests can be administered individually by a psychologist or trained test administrator, or administered on a group basis (Raven, 1998).

#### Verbal working memory task

Computerized forward and backward digit span tests were used to assess verbal WMC, as in our previous study [45]. Subjects were asked to view a progressively increasing number of random digits visually presented one-digit per second on a computer screen. They were then asked to repeat the sequence by pressing numbered buttons on the screen in the presented order (digit-span forward) or in the reverse order (digit-span backward), starting from two digits. Three sequences were given at each level, until the participants responded incorrectly to all three sequences, at which point the task was ended. The score of each test is equal to the sum of the number of digits correctly repeated in the digit span forward and digit span backward tasks.

#### Visuospatial working memory task

A (computerized) visuospatial WM task [46]. In the visuospatial WM task, circles were presented one by one at a rate of 1/s in a four-by-four grid-like interface. Participants had to remember the location and order of the stimuli. After the presentation of stimuli, participants indicated the location and order of the presented stimuli by clicking the grid-like interface on a computer screen with a mouse in the stimuli’s presented order (forward visuospatial WM task) or in the reverse order (backward visuospatialWMtask).

The number of items to be remembered started with two items and progressively increased. Three sequences were given at each level, until the participants responded incorrectly to all three sequences, at which point the task was ended. The score of each test was equal to the sum of the number of items correctly repeated in both the forward visuospatial WM task and the backward visuospatial WM task.

### Image acquisition

All MRI data were acquired using a 3-T Philips Intera Achieva scanner (Philips Medical Systems, Best, The Netherlands). High-resolution T1-weighted structural images (240 × 240 matrix; repetition time, 6.5 ms; echo time, 3 ms; field of view, 24 cm; 162 slices; slice thickness, 1.0 mm) were collected using a magnetisation-prepared rapid gradient echo (MPRAGE) sequence [47], [48].

### Pre-processing of structural data

Pre-processing of structural data was performed using the Statistical Parametric Mapping software (SPM8; Wellcome Department of Cognitive Neurology, London, UK) implemented in MATLAB (Mathworks Inc., Natick, MA, USA). Using the new segmentation algorithm implemented in SPM8, T1-weighted structural images of each individual were segmented into six tissues. In this process, the grey matter tissue probability map (TPM) was manipulated using maps implemented in the software so that the signal intensity of voxels with (grey matter tissue probability of the default tissue grey matter TPM + white matter tissue probability of the default TPM) > 0.25 became 0. When this adjusted grey matter TPM is used, the dura matter is less likely to be classified as grey matter (compared with when the default grey matter TPM is used) without other substantial segmentation problems. In this new segmentation process, default parameters were used, except that affine regularisation was performed with the International Consortium for Brain Mapping template for East Asian brains. We then proceeded to the diffeomorphic anatomical registration through exponentiated lie algebra (DARTEL) registration process implemented in SPM8. In this process, we used DARTEL import of images of the five grey matter TPMs from the abovementioned new segmentation process. First, the template for the DARTEL procedures was created using imaging data from 63 subjects who participated in an experiment in our laboratory [46]. Using this existing template, the DARTEL procedures were performed for all of the subjects in the present study. In these procedures, default parameter settings were used. The resulting images were spatially normalised to the Montreal Neurological Institute (MNI) space to produce images with 1.5 × 1.5 × 1.5 mm^3^ voxels. Additionally, we performed volume change correction (modulation) by modulating each voxel with the Jacobian determinants derived from spatial normalisation, which allowed us to determine regional differences in the absolute amount of brain tissue [49]. Then, all images were smoothed by convolving them with an isotropic Gaussian kernel of 8 mm full width at half maximum for the reasons described below.

### Statistical analysis

The behavioural data were analysed using the statistical software SPSS 20.0 (SPSS Inc., Chicago, IL, USA). Associations between psychological variables were analysed using Pearson’s correlation analysis. Moreover, we performed three different multiple regression analyses (non-voxelwise analyses) in which the dependent variable was tWMV and age, sex and the performance on each cognitive test were independent variables.

First, we assessed rWMV associated with individual differences in simple processing speed task, word–colour and colour–word tasks (processing speed tasks with words) and simple arithmetic task. Statistical analyses of morphological data were performed using the VBM8 software, an extension of SPM8.

In the analyses, we included only voxels that showed rWMV > 0.1 in all subjects. The primary purpose for using white matter thresholds was to cut the periphery of the white matter areas and effectively limit the areas for analyses. We performed this procedure by limiting the areas for analyses to those likely to be white matter. The voxels outside the brain areas are more likely to be affected by signals outside the brain through smoothing. Masking the analysis to brain areas was performed in fMRI analyses of SPM8 by default.

With the whole brain data, we performed three separate multiple regressions for regression analyses to test the relationship between the following: a) simple processing speed and rWMV, b) processing speed tasks with words and rWMV and c) simple arithmetic speed and rWMV. The analyses were performed with sex and age as additional covariates. According to our hypothesis, we performed two analyses: First, we did not use tWMV as a covariate because we were wanted to test whether processing speed globally (non-specifically) correlates with WMV. Second, we analysed the data using tWMV as a covariate to evaluate regional differences in the relationship of WMV with processing speed.

The voxel level threshold was set to P = 0.05 [corrected for false discovery rate (FDR)]. Multiple comparison correction was performed using the FDR approach [50]. FDR-based methods have been shown to be more powerful and sensitive than other available approaches to multiple statistical testing [50–51]).

Finally, anatomical labelling of significant areas was performed using the ICBM-DTI-81 Atlas [52].

We analysed data only for the participants who completed the tasks. This amounted to the data from 831, 887 and 883 participants for the simple processing speed task, simple arithmetic task and Stroop task, respectively.

## Results

Mean (M) score and SD of behavioural data are shown in Table 1. The results of correlation analysis are also shown in Table 1. The processing speed task significantly and positively correlated with the simple arithmetic task (P = 0.0001, r = 0.360), the Stroop word–colour task (P < 0.0001, r = 0.515), the Stroop colour–word task (P < 0.0001, r = 0.578), the RAPM (P < 0.0001, r = 0.340), the Verbal working memory task (P < 0.001, r = 0.242) and Visuospatial working memory task (P < 0.0001, r = 0.332). The simple arithmetic speed task significantly and positively correlated with the Stroop word–colour task (P < 0.0001, r = 0.457),Stroop colour–word task (P < 0.0001, r = 0.384), the Verbal working memory task (P < 0.001, r = 0.203) and the Visuospatial working memory task (P < 0.001, r = 0.137). The simple arithmetic speed task did not correlate with the RAPM (P = 0.893, r = 0.005).

**Table 1:** Pearson's correlation among Processing speed task, Simple arithmetic task, Stroop Word–Colour task and Stroop Colour–Word task, and means (M) and standard deviations (SD).

	1	2	3	4	5	6	7
**1.** Processing speed task	—						
**2.** Simple arithmetic task	.360***	—					
**3.** Stroop Word–Colour task	.515***	.457***	—				
**4.** Stroop Colour–Word task	.578***	.384***	.620***	—			
**5.** Raven's Advanced Progressive Matrix	.340***	.005	.162**	.204**	—		
6. Verbal working memory task	.242**	.203**	.208**	.227**	.293**	—	
**7.** Visuospatial working memory task	.332***	.137**	.172**	.169**	.363***	.372***	—
**M**	49.3	31.4	70.7	52.3	28.1	35.7	28.3
**SD**	7.1	5.3	7.4	6.7	4.9	7.1	4.4

**p < .001

***p < .0001

The Stroop word–colour task significantly and positively correlated with the Stroop colour–word task (P < 0.0001, r = 0.620; Table 1), the RAPM (P < 0.001, r = 0.162), the Verbal working memory task (P < 0.001, r = 0.208) and the Visuospatial working memory task (P < 0.001, r = 0.172). The Stroop Colour–Word task significantly and positively correlated with the RAPM (P < 0.001, r = 0.204), the Verbal working memory task (P < 0.001, r = 0.227) and the Visuospatial working memory task (P < 0.001, r = 0.169). Raven's Advanced Progressive Matrix (RAPM) significantly and positively correlated with the Verbal working memory task (P < 0.001, r = 0.293) and the Visuospatial working memory task (P < 0.0001, r = 0.363). Verbal working memory task significantly and positively correlated with Visuospatial working memory task (P < 0.0001, r = 0.372)Regarding the three multiple regression analyses (non-voxelwise analyses), we verified the relationship between the predictors (cognitive task performance) and the outcome (tWMV), and the relationship was positive and significant (simple processing speed: β = 0.093, P < 0.001, R^2^ = 0.358; Stroop task: β = 0.098, P < 0.0001, R^2^ = 0.356; simple arithmetic speed: β = 0.089, P < 0.001, R^2^ = 0.355).

### Correlation of rWMV and the processing speed task

**Fig 1:**
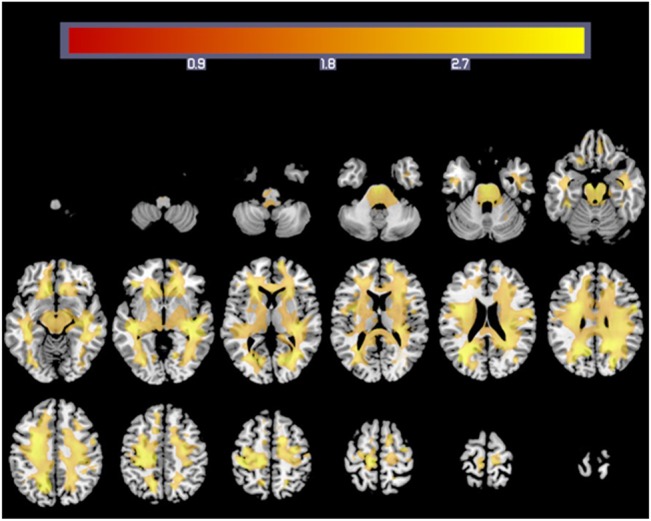
White matter regions showing a correlation between rWMV and Simple Processing Speed task performance. Colourbar indicates the t-values for the regression slopes.

After adjusting for age and sex, multiple regression analysis (FDR correction, P = 0.05) revealed a positive and significant correlation between performance on the simple processing speed task and rWMV across the whole white matter areas (Fig 1 and Table 2). Only in the left uncinate fasciculus we did not find a correlation between rWMV and performance on the simple processing speed task.

**Table 2:** Brain regions of significant correlation between rWMV and Simple Processing Speed task.

Region	N of significant voxel (Total N of voxel)	Peak t-value	Corrected p value (FDR)	MNI peak coordinates		
				x	y	z
Middle cerebellar peduncle	1691 (5391)	3.60	0.007	-2	-16	-32
Pontine crossing tract	424 (522)	3.36	0.010	0	-21	-24
Genu of corpus callosum	2036 (3077)	3,25	0.011	-14	24	-8
Body of corpus callosum	2785 (4759)	3.31	0.010	-20	3	31
Splenium of corpus callosum	2923 (4182)	4.3	0.004	-23	-52	-22
Fornix	5 (271)	2.74	0.019	-3	-21	18
Corticospinal tract right	350 (495)	3.31	0.010	-5	-21	-33
Corticospinal tract left	317 (497)	3.37	0.010	5	-18	-26
Medial lemniscus right	198 (265)	2.95	0.015	-5	-34	-26
Medial lemniscus left	204 (264)	3.02	0.014	3	-33	-26
Inferior cerebellar peduncle right	92 (377)	2.31	0.032	-9	-40	-35
Inferior cerebellar peduncle left	81 (367)	2.21	0.36	9	-40	-33
Superior cerebellar peduncle right	140 (372)	2.90	0.016	-6	-34	-24
Superior cerebellar peduncle left	144 (379)	3.04	0.014	5	-33	-23
Cerebral peduncle right	654 (833)	3.21	0.012	-6	-18	-21
Cerebral peduncle left	717 (827)	3.19	0.012	5	-16	-21
Anterior limb of internal capsule right	783 (1111)	3.52	0.008	-23	21	-0631
Anterior limb of internal capsule left	631 (1086)	3.16	0.012	21	21	3
Posterior limb of internal capsule right	943 (1320)	2.60	0.022	-26	-6	18
Posterior limb of internal capsule left	1026 (1312)	3.16	0.012	27	-27	-18
Retrolenticular part of internal capsule right	672 (881)	3.67	0.007	-41	-27	-3
Retrolenticular part of internal capsule left	739 (859)	3.92	0.006	36	-24	-3
Anterior corona radiata right	1571 (2368)	3.70	0.007	-12	29	-12
Anterior corona radiata left	1987 (2338)	3.35	0.010	15	39	-3
Superior corona radiata right	2177 (2448)	4.33	0.004	-26	3	34
Superior corona radiata left	2098 (2479)	3.07	0.014	24	-4	37
Posterior corona radiata right	1018 (1301)	4.58	0.003	-27	-57	22
Posterior corona radiata left	1102 (1305)	4.07	0.005	21	-52	28
Posterior thalamic radiation (include optic radiation) right	926 (1376)	4.13	0.005	-33	-57	18
Posterior thalamic radiation (include optic radiation) left	1044 (1386)	3.69	0.007	35	-63	3
Sagittal stratum right	625 (773)	3.76	0.006	-42	-27	-6
Sagittal stratum left	602 (783)	4.03	0.005	39	-21	-6
External capsule right	518 (1329)	3.49	0.008	-24	21	-0
External capsule left	713 (1299)	3.55	0.008	33	-19	-3
Cingulum (cingulate gyrus) right	111 (923)	2.98	0.015	-11	-37	-33
Cingulum (cingulate gyrus) left	440 (1086)	2.97	0.015	9	-27	30
Cingulum (hippocampus) right	10 (467)	2.18	0.037	-12	-46	9
Cingulum (hippocampus) left	51 (452)	2.39	0.029	20	-25	-21
Fornix (cres) / Stria terminalis right	232 (435)	2.79	0.018	-32	-30	-5
Fornix (cres) / Stria terminalis left	311 (426)	3.82	0.006	32	-24	-6
Superior longitudinal fasciculus right	45 (2340)	4.22	0.004	-36	-58	19
	1094	3.88	0.007	-32	2	28
Superior longitudinal	1756 (2332)	3.52	0.008	29	-48	30
fasciculus left						
Superior fronto-occipital fasciculus right	119 (175)	3.17	0.012	-23	0	24
Superior fronto-occipital fasciculus left	125 (165)	2.63	0.022	21	12	19
Inferior fronto-occipital fasciculus right	168 (693)	3.25	0.011	-21	21	-5
Inferior fronto-occipital fasciculus left	74 (638)	3.62	0.007	35	-19	-16
Uncinate fasciculus right	-	-	-	-	-	-
Uncinate fasciculus left	39 (150)	2.50	0.025	36	-4	-18
Tapatum right	77 (219)	3.42	0.009	-30	-52	15
Tapatum left	161 (239)	3.23	0.011	24	-46	22

In contrast, after adjusting for age, sex and tWMV, multiple regression analysis revealed that performance on the processing speed task did not correlate with rWMV in any of the regions.

### Correlation of rWMV and the simple arithmetic task

**Fig 2:**
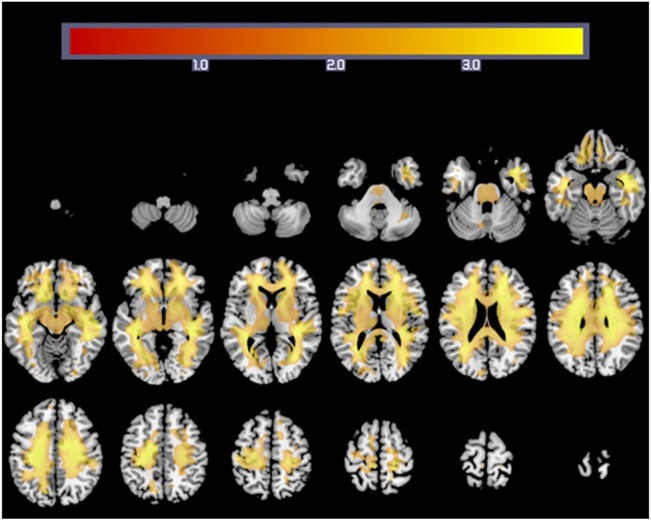
White matter regions showing a correlation between rWMV and Simple arithmetic task performance. Colourbar indicates the t-values for the regression slopes.

After adjusting for age and sex, multiple regression analysis (FDR correction, P < 0.05) revealed a positive and significant correlation between performance on the simple arithmetic task and rWMV across the whole white matter areas (Fig 2 and Table 3). Only in the left inferior cerebellar peduncle, the fornix, the left cingulum (hippocampus) and the right uncinate fasciculus we did not find a correlation between WMV and performance on the simple arithmetic task.

**Table 3:** Brain regions of significant correlation between rWMV and Simple arithmetic task.

Region	N of significant voxel (Total N of voxel)	Peak t-value	Corrected p value (FDR)	MNI peak coordinates		
				x	y	z
Middle cerebellar peduncle	606 (5391)	2.58	0.015	-2	-15	-30
Pontine crossing tract (a part of MCP)	348 (522)	2.47	0.018	-2	-24	-23
Genu of corpus callosum	2601 (3077)	4.00	0.003	18	24	22
Body of corpus callosum	4076 (4759)	4,56	0.003	12	-6	36
Splenium of corpus	2978 (4182)	4.08	0.003	-29	-57	9
callosum	75	3.45	0.003	26	-58	12
Fornix	-	-	-	-	-	-
Corticospinal tract right	273 (495)	2.46	0.019	-9	-22	-23
Corticospinal tract left	270 (497)	2.37	0.022	5	-24	-23
Medial lemniscus right	149 (265)	2.22	0.028	-5	-33	-26
Medial lemniscus left	113 (264)	2.21	0.028	3	-33	-26
Inferior cerebellar peduncle right	7 (377)	1.91	0.048	-8	-42	-41
Inferior cerebellar peduncle left	-	-	-	-	-	-
Superior cerebellar peduncle right	87 (372)	2.29	0.025	-5	-30	-20
Superior cerebellar peduncle left	94 (379)	2.55	0.016	6	-33	-15
Cerebral peduncle right	654 (833)	2.56	0.016	-12	-15	-18
Cerebral peduncle left	678 (827)	2.70	0.012	12	-6	-5
Anterior limb of internal capsule right	889 (1111)	3.43	0.003	-21	12	18
Anterior limb of internal capsule left	882 (1086)	3.75	0.003	20	18	16
Posterior limb of internal capsule right	348 (1320)	2.47	0.018	-2	-24	-23
Posterior limb of internal capsule left	1029 (1312)	3.06	.006	24	-6	18
Retrolenticular part of internal capsule right	722 (881)	4.28	0.003	-36	-33	-3
Retrolenticular part of internal capsule left	720 (859)	4.30	0.003	38	-30	-3
Anterior corona radiata right	2035 (2368)	3.68	0.003	-21	14	27
Anterior corona radiata left	2035 (2338)	4.27	0.003	17	27	25
Superior corona radiata right	2206 (2448)	4.43	0.003	-26	2	30
Superior corona radiata left	2218 (2479)	4.20	0.003	17	-6	36
Posterior corona radiata right	1151 (1301)	4.10	0.003	-32	-55	19
Posterior corona radiata left	1071 (1305)	3.99	0.003	21	-30	30
Posterior thalamic radiation (include optic radiation) right	1212 (1376)	4.12	0.003	-32	-55	18
Posterior thalamic radiation (include optic radiation) left	1228 (1386)	3.85	0.003	30	-66	3
Sagittal stratum right	625 (773)	4.32	0.003	-38	-33	-5
Sagittal stratum ùleft	625 (783)	4.28	0.003	38	-30	-5
External capsule right	82 (1329)	3.50	0.003	-36	-16	-3
	535	3.18	0.005	-27	8	18
External capsule left	915 (1299)	3.59	0.003	26	9	18
Cingulum (cingulate gyrus) right	565 (923)	3.15	0.005	-12	-36	33
Cingulum (cingulate gyrus) left	674 (1086)	4.52	0.003	9	-4	37
Cingulum (hippocampus) right	8 (467)	2.19	0,030	-9	-45	7
Cingulum (hippocampus) left	-	-	-	-	-	-
Fornix (cres) / Stria terminalis right	298 (435)	3.39	0.004	-35	-15	-11
Fornix (cres) / Stria terminalis left	227 (426)	3.32	0.004	35	-12	-17
Superior longitudinal fasciculus right	1883 (2340)	4.35	0.003	-30	-24	40
Superior longitudinal fasciculus left	1930 (2332)	3.90	0.003	2	-12	36
Superior fronto-occipital fasciculus right	125 (175)	4.01	0.003	-21	5	24
Superior fronto-occipital fasciculus left	125 (165)	3.76	0.003	20	17	19
Inferior	181 (693)	3.90	0.003	-18	14	-12
fronto-occipital fasciculus right	166	3.70	0.003	-38	-15	-8
Inferior	251 (638)	3.25	0.004	36	-12	-12
fronto-occipital fasciculus left	137	3.19	0.005	17	14	-12
Uncinate fasciculus right	-	-	-	-	-	-
Uncinate fasciculus left	111 (150)	3.57	0.003	36	-1	-21
Tapatum right	183 (219)	3.90	0.003	-32	-52	15
Tapatum left	154 (239)	3.30	0.004	29	-54	7

Additionally, in this case, after adjusting for age, sex and tWMV, multiple regression analysis revealed that performance on the simple arithmetic task did not correlate with rWMV in any of the regions.

#### Correlation of rWMV and Stroop task

**Fig 3:**
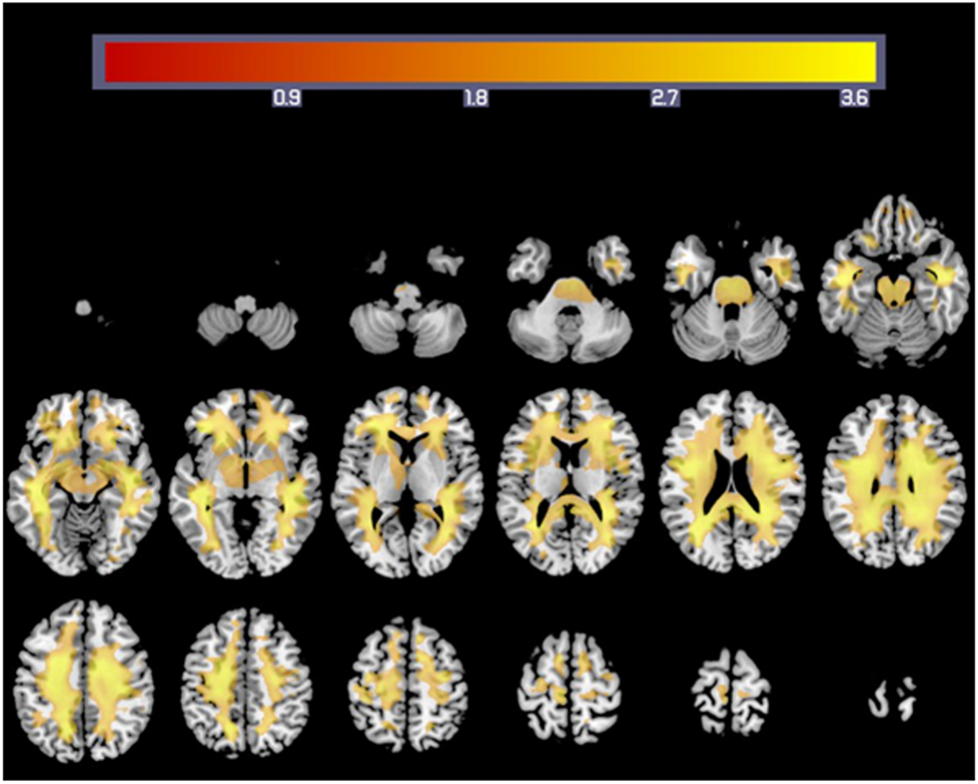
White matter regions showing a correlation between rWMV and Stroop task performance. Colourbar indicates the t-values for the regression slopes.

First, we created a normalised sum of scores on the word–colour and colour–word tasks. After adjusting for age and sex, multiple regression analysis (FDR correction, P < 0.05) showed a positive and significant correlation between performance on the Stroop task and rWMV across the whole white matter areas (Fig 3 and Table 4). Furthermore, in this case, we did not find a correlation between performance on the Stroop task and rWMV in the left and right inferior cerebellar peduncle and in the left cingulum (hippocampus). Moreover, after adjusting for age, sex and tWMV, multiple regression analysis showed that performance on the Stroop task did not correlate with rWMV in any of the regions.

**Table 4:** Brain regions of significant correlation between rWMV and Stroop task.

Region	N of significant voxel (Total N of voxel)	Peak t-value	Corrected p value (FDR)	MNI peak coordinates		
				x	y	z
Middle cerebellar peduncle	1231 (5391)	3.30	0.006	2	-19	-27
Pontine crossing tract (a part of MCP)	376 (522)	3.27	0.006	0	-21	-24
Genu of corpus callosum	1994 (3077)	3.48	0.005	17	20	25
Body of corpus callosum	2881 (4759)	4.55	0.004	14	-3	37
Splenium of corpus callosum	3542 (4182)	4.43	0.004	-27	-54	19
Fornix	17 (271)	2.45	0.022	-2	3	1
Corticospinal tract right	313 (495)	3.20	0.006	-6	-18	-26
Corticospinal tract left	312 (497)	3.26	0.006	5	-19	-29
Medial lemniscus right	72 (265)	2.54	0.019	-5	-33	-26
Medial lemniscus left	85 (264)	2.54	0.019	3	-33	-26
Inferior cerebellar peduncle right	-	-	-	-	-	-
Inferior cerebellar peduncle left	-	-	-	-	-	-
Superior cerebellar peduncle right	81 (372)	2.55	0.018	-6	-30	-21
Superior cerebellar peduncle left	95 (379)	2.68	0.015	5	-30	-21
Cerebral peduncle right	670 (833)	2.96	0.009	-6	-19	-21
Cerebral peduncle left	550 (827)	2.97	0.009	5	-21	-21
Anterior limb of internal capsule right	744 (1111)	3.38	0.005	-23	21	-0
Anterior limb of internal capsule left	372 (1086)	3.14	0.007	21	15	18
Posterior limb of	186 (1320)	2.43	0.023	-14	-3	-2
internal capsule right	97	2.41	0.024	-26	-6	18
Posterior limb of	108 (1312)	2.63	0.016	27	-27	18
internal capsule left	33	2.07	0.041	12	-4	-3
Retrolenticular part of internal capsule right	568 (881)	4.46	0.004	-41	-30	-3
Retrolenticular part of internal capsule left	595 (859)	3.76	0.004	41	-36	-3
Anterior corona radiata right	2013 (2368)	3.80	0.004	-27	35	3
Anterior corona radiata left	2004 (2338)	3.68	0.004	15	17	30
Superior corona radiata right	2206 (2448)	4.61	0.004	-24	-1	37
Superior corona radiata left	2209 (2479)	4.49	0.004	14	-3	39
Posterior corona radiata right	1151 (1301)	4.53	0.004	-30	-55	21
Posterior corona radiata left	1132 (1305)	4.19	0.004	27	-43	21
Posterior thalamic radiation (include optic radiation) right	1197 (1376)	4.46	0.004	-30	-55	18
Posterior thalamic radiation (include optic radiation) left	1221 (1386)	4.07	0.004	29	-45	18
Sagittal stratum ùright	625 (773)	4.80	0.004	-42	-30	-6
Sagittal stratum ùleft	617 (783)	3.80	0.004	39	-12	-17
External capsule right	406 (1329)	3.48	0.005	-24	21	-3
	36	2.62	0.016	-35	-22	1
External capsule left	477 (1299)	3.19	0.007	27	9	18
	36	2.55	0.018	33	-22	1
Cingulum (cingulate gyrus) right	256 (923)	3.64	0.004	-9	-34	33
	61	2.34	0.027	-11	9	31
	23	2.17	0.035	-9	32	12
Cingulum (cingulate gyrus) left	800 (1086)	4.02	0.00	9	-6	36
Cingulum (hippocampus) right	19 (467)	2.50	0.020	-12	-46	9
Cingulum (hippocampus) left	-	-	-	-	-	-
Fornix (cres) / Stria terminalis right	182 (435)	3.07	0.008	-36	-12	-17
Fornix (cres) / Stria terminalis left	141 (426)	3.26	0.006	33	-7	-18
Superior longitudinal fasciculus right	1659 (2340)	4.33	0.004	-36	-1	27
Superior longitudinal fasciculus left	1946 (2332)	3.97	0.004	33	2	27
Superior fronto-occipital fasciculus right	125 (175)	3.40	0.005	-23	0	24
Superior fronto-occipital fasciculus left	117 (165)	3.50	0.005	20	9	24
Inferior fronto-occipital fasciculus right	450 (693)	3.4	0.005	-24	21	-5
Inferior fronto-occipital fasciculus left	424 (638)	3.16	0.007	23	21	-6
Uncinate fasciculus right	43 (145)	2.50	0.020	-35	-4	-14
Uncinate fasciculus left	109 (150)	3.40	0.005	36	-4	-18
Tapatum right	183 (219)	4.02	0.004	-30	-52	15
Tapatum left	167 (239)	4.18	0.004	24	-45	22

## Discussion

To the best of our knowledge, this is the first study to explore the associations between rWMV and processing speed tasks in healthy adults at the whole brain level. First, as in previous studies [2], [6], [7], correlation analyses showed a positive correlation between processing speed tasks and working memory and general intelligent tasks. This result are on line with the literature, considering that more are complex the processing speed tasks, stronger is the relationship between processing speed and intelligence and vice versa [53]. Second, VBM analyses showed a positive relationship across the whole white matter between WMV and processing speed performance. Our results support the assumption that WMV is globally related to processing speed performance regardless of the type of processing speed tasks. However, in our results some WM area are not correlated with the processing speed performances. This could be possible considering the high specialization and the lateralization of this areas that consequently they are not involved in processing speed activity. These results do not support the other assumption, i.e., processing speed performance is related to volume of a specific white matter area.

Considering the physiological functions of white matter, we found evidence that WMV might be essential for processing speed, which is often viewed as a key variable of cognitive architecture[2], [6]. In fact, processing speed in each task is likely to largely depend on the properties of white matter that are essential for performance on that task [5–46]. White matter consists mostly of glial cells and myelinated axons; it controls the signals that neurons share, coordinating the cooperative work of brain regions [52]. In fact, the speed of neural signals is associated with the thickness and degree of myelination of axons [16], [54], [55]. As we previously discussed [29], [42], enhancement of these physiological components, which is presumably secondary to increased myelination [56] or increased axonal calibre [57], may be associated with greater effectiveness of neural circuit communication and consequently may facilitate cognitive functions [47].

Our results suggest that globally distributed white matter structures support performance on different processing speed tasks, and this finding is in agreement with the following studies. Previous psychological studies showed that processing speed correlates with performance in various cognitive domains during development and healthy ageing [58], [59]. Furthermore, general white matter integrity is considered a lifelong stable biological foundation of processing speed throughout the lifespan [2], [15], [17]. Simultaneously, performance on so-called processing speed tasks is actually considered to depend on various cognitive activities. For example, the digit symbol test [8], which is a typical test of processing speed, is considered to be affected by psychomotor speed, attention, perceptual organisation, motor persistence and visual short-term memory [60], [61]. Thus, various white matter structures of brain areas may be responsible for processing speed. We may assume that processing speed is a fundamental component of cognitive efficiency or cognitive proficiency, and our results show that data from global WMV partly support this idea and help to explain that variable.

The present findings are expected to stimulate further research in this area, in particular how the relationship between processing speed and WMV can change during cognitive development and ageing process. Moreover, it seems important to test how the relationship between rWMV and improvements in processing speed via training [46] changes in healthy people and patients with cognitive impairment.

This study has some limitations. One limitation is shared with our previous studies and studies by others that involve college cohorts [39], [44], [47], [62], [63]. As mentioned above, we tested young healthy subjects at a relatively high educational level. Limited sampling of the full range of intellectual abilities is a common problem when sampling from college cohorts [57]. Limited sampling may be an important step to rule out the effects of age or the education level that could strongly influence brain structures and increase sensitivity of the analyses. In fact, processing speed correlates with age-related changes in cognition in the course of childhood development [15], [64], [65] (and healthy ageing [2], [15]

This study seems to be the first study to explore the associations between rWMV and processing speed in a large sample of young adults. Supporting our hypothesis, the results confirm that WMV is related globally to performance on different tests of processing speed. Our results support the notion that global WMV could help to explain in detail the influence of white matter on processing speed performance.

## Supporting Information

S1 Table Psychological data of the included participants (Psychological_data_SPSS_File_The relationship between processing speed and regional white matter volume in healthy young people.sav). (SAV)
